# Nintedanib in the prevention of radiation-induced lung injury in patients with unresectable stage II-III NSCLC: A protocol for a randomized, multicenter, control group study

**DOI:** 10.1371/journal.pone.0356205

**Published:** 2026-09-03

**Authors:** Ai Cui, Anhui Shi, Lei Deng, Yanyan Sun, Jiayi Yu, Xinrong Lian, Ziyue Zhao

**Affiliations:** 1 Department of Pulmonary and Critical Care Medicine, Beijing Chao-Yang Hospital, Capital Medical University, Beijing, P.R. China; 2 Department of Respiratory and Critical Care Medicine, The First Hospital of Zhangjiakou, Zhangjiakou, Hebei, P.R. China; 3 Department of Radiation Oncology, Beijing Cancer Hospital, Beijing, P.R. China; 4 Department of Radiation Oncology, Cancer Hospital Chinese Academy of Medical Sciences, Beijing, P.R. China; Showa University Fujigaoka Hospital, JAPAN

## Abstract

**Background:**

Radiation-induced lung injury (RILI) is a common and dose-limiting complication in patients with non-small cell lung cancer (NSCLC) receiving radiotherapy, especially in combination with chemotherapy and/or immunotherapy. RILI encompasses acute radiation pneumonitis (RP) and chronic pulmonary fibrosis, significantly impacting patients’ quality of life and treatment outcomes. Preclinical and clinical evidence suggests that nintedanib, a tyrosine kinase inhibitor approved for idiopathic pulmonary fibrosis (IPF), may have preventive effects against RILI due to shared fibrotic pathways. However, prospective data on its prophylactic use remain limited.

**Methods:**

This is a randomized, multicenter, unblinded, controlled trial designed to evaluate the efficacy and safety of nintedanib in preventing RILI. A total of 66 patients with unresectable stage II-III NSCLC scheduled to receive CCRT will be enrolled from three centers in China. Eligible participants will be randomized 1:1 to either the nintedanib group or the control group. Randomization is stratified by concurrent chemotherapy regimen (etoposide-based vs. taxane-based vs. pemetrexed-based). The nintedanib group will receive oral nintedanib 150 mg twice daily, initiated 1–2 weeks before radiotherapy and continued for 12 weeks, followed by a voluntary treatment period from week 13 to week 24. The control group will receive CCRT alone without nintedanib. All patients will undergo standardized CCRT comprising platinum-based doublet chemotherapy (cisplatin or carboplatin combined with etoposide, taxanes, or pemetrexed) and radiotherapy (60 Gy/30 fractions). Immunotherapy consolidation is permitted as a viable treatment option per guideline recommendations. Primary endpoint: The incidence of grade ≥2 RILI (according to CTCAE v5.0) within 6 months after radiotherapy initiation. Secondary endpoints: Time to radiological lung injury; changes in pulmonary function (FVC%, FEV1%, DLco%); tumor response outcomes (OS, PFS, ORR); safety and tolerability; and patient-reported outcomes. This trial will provide the first prospective evidence on the efficacy of nintedanib for preventing RILI in patients with unresectable stage II-III NSCLC undergoing CCRT. The results may inform clinical practice and guide future prophylactic strategies for RILI.

**Trial registration:**

This study was registered on April 16, 2025, at the Chinese Clinical Trial Registry (No. ChiCTR2500100845).

**Reporting guideline:**

This protocol was developed in accordance with the SPIRIT (Standard Protocol Items: Recommendations for Interventional Trials) guidelines.

## Introduction

Lung cancer is the leading cause of morbidity and mortality among all types of malignant tumors. In 2022, there were 2.48 million new cases of lung cancer reported globally, representing 12.5% of all new cancer cases worldwide. China accounted for 1.06 million of these new cases. Adenocarcinoma is the most common pathological type of lung cancer, comprising over 50% of cases, while squamous carcinoma is the second most common type, with an incidence rate of approximately 30%. Lung cancer ranks highest in global cancer mortality, causing an estimated 1.8 million deaths in 2022, representing 18.7% of all cancer-related deaths worldwide [1,2]. The high incidence and poor prognosis of lung cancer make it a significant public health concern worldwide.

The utilization of radiotherapy (RT), which employs high-energy radiation to target and eradicate cancer cells selectively, is frequently used as a primary therapeutic modality for lung cancer, especially in individuals with locally advanced disease or in those who are not candidates for surgery [3]. RT can also be used in combination with surgery or chemotherapy to improve outcomes and reduce the risk of cancer recurrence. Radiation-induced lung injury (RILI), consisting of early radiation pneumonitis (RP) and late radiation pulmonary fibrosis (RPF), is a common condition that significantly impacts the quality of life of patients with lung cancer receiving RT [4,5]. RP usually presents within 2–6 months following CRRT. The incidence of symptomatic (grade ≥2) radiation pneumonitis in patients receiving CCRT for locally advanced NSCLC is approximately 10%–20%, with severe (grade ≥3) cases affecting 10%–20% and carrying a mortality rate of up to 50% [6,7]. Although consolidation durvalumab after CCRT significantly improves survival [8], it increases pneumonitis risk (33.9% vs. 24.8% with radiotherapy alone) [8]. Pneumonitis often leads to interruption or discontinuation of immunotherapy, underscoring the need for effective preventive strategies to ensure patients derive full benefit from both treatments. The dose-response relationship in radiation oncology aims to optimize treatment outcomes while considering the potential for increased harm to healthy tissues and emphasizes the significance of comprehending and controlling RILI to reduce its effects on patients receiving RT [9].

The clinical manifestations of RILI exhibit a range of symptoms such as cough, fever, and dyspnea [10]. These symptoms can vary in severity and may present differently in different individuals. Pathological imaging during the initial stages reveals infiltrative alterations. As the disease progresses, different patterns develop in the irradiated region, including patchy opacities, indications of ventilated bronchi, and honeycomb-like appearances. Some patients may also show changes beyond the irradiated field, along with modifications within the treated region [11–14]. A gradual onset of lung fibrosis is a characteristic feature of RPF development, with symptoms often appearing between six months to several years after CRRT. Imaging results in RPF commonly show changes of interstitial alterations, including decreased lung capacity, increased shadowing from fibrous tissue, thickening of tissue within interlobular septa, thickening of the pleura, and displacement of structures in the chest cavity. The development of RILI involves an intricate interaction between damage caused by radiation and the body’s immune reactions. Prognostic factors influencing the development of RP encompass the dose of radiotherapy, volume of lung tissue exposed to radiation, baseline lung function, and concurrent use of chemotherapeutic or immunologic medications.

In clinical practice, RILI is typically treated with glucocorticoids and supportive therapies like oxygen therapy and infection management [15]. The interventions are focused on relieving symptoms and aiding in the patient’s recovery from RILI. However, the clinical outcomes of RILI remain unsatisfactory, suggesting that preventive measures may be more crucial than treatment in managing RILI. Currently, strategies to prevent radiological lung injury after radiotherapy in patients with unresectable NSCLC include optimizing radiotherapy techniques, adjusting treatment regimens, and employing other adjuvant measures. However, further studies and clinical trials are still needed to validate the efficacy and safety of these approaches and to find more effective preventive drugs.

Studies have indicated that RILI and idiopathic pulmonary fibrosis (IPF) share similar pathological mechanisms [16–19], implying that interventions targeting shared fibrotic pathways may be beneficial for both conditions. This finding highlights the potential for novel treatment approaches for RILI by focusing on common mechanisms underlying fibrosis. Nintedanib is a small molecule inhibitor that targets multiple tyrosine kinases including platelet-derived growth factor receptor, fibroblast growth factor receptor, vascular endothelial growth factor receptor, and Fms-like tyrosine kinase-3. The compound competitively binds to the ATP-binding sites of lung fibroblasts, leading to the inhibition of their proliferation, migration, and transformation [20]. Also, nintedanib has demonstrated anti-inflammatory and enhanced vascular remodeling effects in pulmonary fibrosis. The mechanisms of action of nintedanib contribute to the reduction of pulmonary fibrosis progression and the enhancement of lung function and quality of life in affected individuals [21]. There is still a lack of published clinical data on the preventive effects of nintedanib in RILI.

This study is a randomized, controlled, multicenter clinical trial that aims to examine the effectiveness of nintedanib, compared to a control group not receiving nintedanib, in preventing RILI in patients with unresectable stage II-III non-small cell lung cancer (NSCLC) undergoing concurrent chemoradiation therapy (CCRT).

## Materials and methods

### Trial objectives and endpoints

The primary objective of this trial is to assess the efficacy of nintedanib in preventing RILI by comparing the incidence of grade 2 or higher radiation pneumonitis according to CTCAE grading between the two groups of patients with unresectable stage II or III NSCLC following CRRT. The secondary objectives of the study included comparing time to radiological lung injury occurrence; pulmonary function changes after radiotherapy; treatment effects in patients with RILI; the efficacy outcomes (OS, PFS, and ORR) in patients with NSCLC; and safety as well as tolerability.

The study focused on clinically meaningful events such as RP occurrence and therapeutic efficacy. Patients reported outcomes included changes in symptoms like shortness of breath, cough, and fatigue, as well as the impact on daily life and overall health status assessment at different time points, which will be compared to baseline. Additionally, lung function (FVC%, FEV1%, DLco%) and HRCT exploratory assessment were evaluated at various time points to compare with the baseline measurements.

### Trial design and trial population

**Fig 1:**
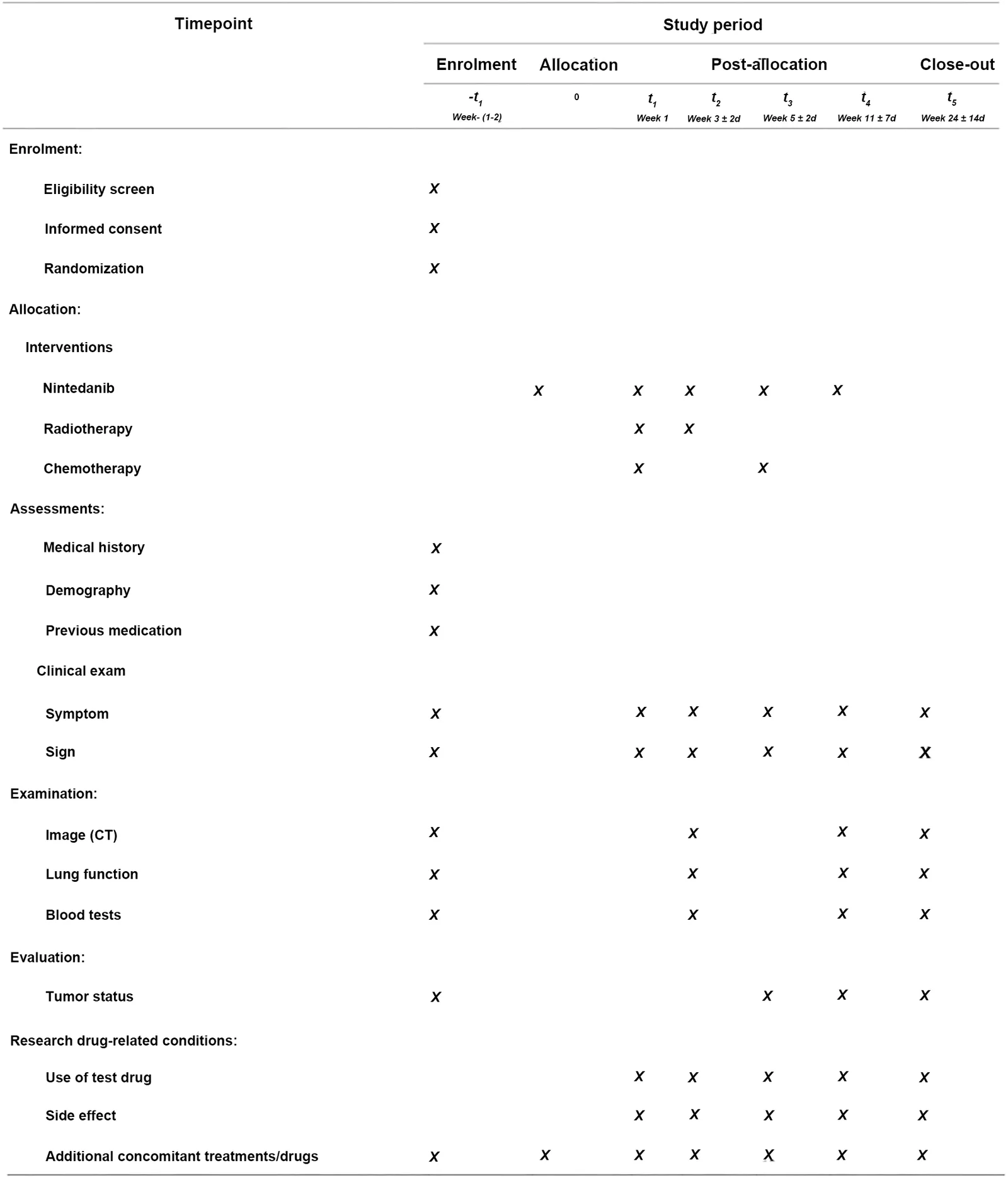
Example template of recommended content for the schedule of enrolment, interventions, and assessments.

**Fig 2:**
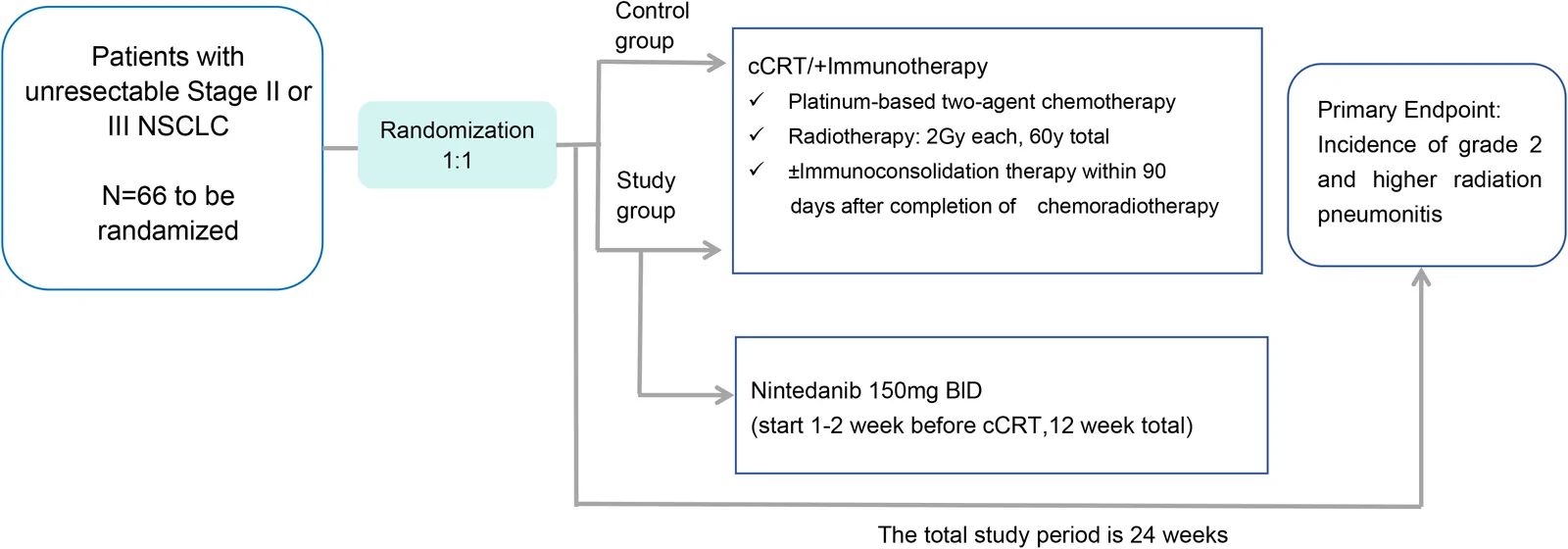
Flowchart of the clinical trial protocol. NSCLC: Non-small-cell lung cancer; cCRT: Concurrent chemoradiotherapy.

This is a prospective, randomized, multicenter, unblinding, control group study aimed at investigating the potential efficacy and safety of nintedanib in preventing RILI in patients with NSCLC undergoing RT. The intervention involves administering nintedanib 1–2 week before CRRT to assess stable blood concentration of the drug at the start of RT. The detailed follow-up program is shown in Fig 1. Fig 2 illustrates the overview of the study procedure.

Eligible participants will be randomised at a 1:1 ratio to the nintedanib group or the control group using a random number generator. Randomization will be stratified by the concurrent chemotherapy regimen, which is categorized into three groups: (1) platinum-based doublet with etoposide; (2) platinum-based doublet with taxanes (docetaxel/paclitaxel); and (3) platinum-based doublet with pemetrexed. Patients receiving vinorelbine will be included in the taxane group due to similar clinical practice patterns and expected sample size considerations. This stratification factor was selected because chemotherapy regimen is one of the strongest predictors of radiation pneumonitis risk, with etoposide-based regimens showing higher pulmonary toxicity compared to pemetrexed-based regimens.

Other potential confounding factors, including radiotherapy planning target volume (PTV), baseline pulmonary function (FVC%/FEV1%), and immunotherapy consolidation (yes/no), will be carefully recorded and adjusted for in the multivariable regression analysis.

Patients diagnosed with unresectable stage II-III NSCLC who comply with trial requirements may qualify to participate in the trial. Non-small cell lung cancer (NSCLC) will be confirmed through histological or cytological examination and is not suitable for surgical removal. The TNM system will be used to classify patients into different stages based on the size of the primary tumor, lymph node involvement, and presence of distant metastasis (the International Association for the Study of Lung Cancer staging manual (8th edition)) [22].

For patients with Stage II NSCLC, eligibility is restricted to those who are medically inoperable or refuse surgery after thorough multidisciplinary discussion. Medical inoperability is defined as the presence of severe comorbidities that preclude surgery, including but not limited to: severe baseline respiratory diseases (e.g., COPD, interstitial lung disease, pulmonary hypertension, et al.), severe cardiac dysfunction (NYHA Class III-IV), or other major medical conditions that contraindicate general anesthesia and surgical resection. Patients with Stage II disease who are surgical candidates but decline surgery after being fully informed of the risks and benefits of both surgical and non-surgical approaches will also be eligible. All Stage II patients will be reviewed by a multidisciplinary team (including a thoracic surgeon, radiation oncologist, and pulmonologist) to confirm inoperability or refusal prior to enrollment.

A total of 66 participants will be enrolled and randomized at 1:1 to nintedanib and control groups through the use of a random number generator. To ensure rigorous allocation concealment, the following standardized procedure will be implemented: potential participants identified in clinical practice will be referred to the study team for formal screening. Upon confirmation of eligibility and completion of informed consent by the study investigators or coordinators, an independent staff member—who is not involved in recruitment or clinical assessments—will assign each enrolled participant to an intervention group. This assignment will be executed through a centralized randomization, which allocates participants according to a pre‑generated sequence.

Table 1 outlines the specific criteria for inclusion, exclusion, and withdrawal in the study. Participants meeting the eligibility criteria should provide written informed and commit to actively taking part in and finishing the study, along with all necessary follow-up procedures. Before the commencement of the trial, all participants are required to undergo a series of examinations and counseling sessions within a timeframe of 1–2 weeks. These procedures are mandatory for each patient.

**Table 1:** Key eligibility criteria for the trial.

Inclusion criteria
• Age between 18 and 80 years at time of screening • Histologically/cytologically confirmed diagnosis of NSCLC • TNM staging of stage II or III according to the International Association for the Study of Lung Cancerstaging manual (8th edition): medically inoperable (defined as severe comorbidities precluding surgery) or refusal of surgery after multidisciplinary consultation. • Unsuitable for or unable to tolerate surgery • WHO performance status of 0 or 1 at randomization • No previous radiation therapy • Be able to swallow gelatin pills • Suitable for completing pulmonary function tests • Volunteered to participate in the study and sign an informed consent form
Exclusion criteria
• Expected life expectancy of <6 months at randomization • Severe cardiovascular, cerebrovascular, neurological diseases, infections, epilepsy, active peptic ulcer, psychological disorders, etc. • Abnormal liver function (transaminases elevated to 2.5 times or more the upper limit of normal) • Moderate or severe hepatic impairment (Child-Pugh B or C) • Abnormal renal function (creatinine clearance <30 ml/min) or requiring dialysis • Recent anticoagulation therapy or combination of deep vein thrombosis, pulmonary embolism, heart attack and cerebral infarction, etc. • Antifibrotic therapy within 1 month • Concurrent use of strong CYP3A4 inhibitors or inducers • Allergy or intolerance to therapeutic drugs and ingredients • Pregnant or breastfeeding women
Withdrawal criteria
• Prolonged treatment shedding exceeding 48 hours or treatment discontinuation • Researchers decided that the patient was not a suitable candidate for radiotherapy as originally planned
Determination of dropout
• Discontinuation of the clinical trial by a subject who, for whatever reason, is unwilling/unable to continue with the trial and requests withdrawal from the trial • Although not explicitly withdrawing from the trial, subjects no longer receive medication and testing and are dropped from the trial • Difficulty in continuing the medication due to the occurrence of other complications or worsening of comorbidities, as judged by the investigator • Experience an adverse drug reaction event that warrants discontinuation of the trial, as judged by the investigator • Other conditions occur that make it difficult to continue the medication, as judged by the investigator

We will employ a multi-center design and conduct comprehensive start-up training for all site teams to ensure full protocol comprehension, supported by a clear enrollment flowchart for streamlined screening. Recruitment progress, screen failure rates, and reasons will be monitored weekly/monthly to enable timely strategy adjustments and ensure target sample size attainment.

This study was registered on April 16, 2025, at the Chinese Clinical Trial Registry (No. ChiCTR2500100845). The authors confirm that all ongoing and related trials for this drug/intervention are registered. This study entered the study phase and subjects began to be enrolled on 01/05/2025 and concluded on 30/10/2027.

### Data collection methods

The Case Report Form (CRF) was used as the main data collection tool in this study. The design of the CRF form was rigorously planned and contained multiple sections that comprehensively covered the subject’s basic information, enrollment information, course of treatment, signs and symptoms, examination and test results, and health-related questionnaires. The specific structure of the CRF form is as follows:

- Basic information: Record the subjects’ name, age, gender, contact information and other basic information.
- Enrollment information: Record the enrollment time, enrollment criteria, exclusion criteria, etc. of the subjects.
- Tumor molecular characteristics: Record the driver mutation status (including but not limited to EGFR, ALK, ROS1, KRAS) if available from routine clinical testing. This information will be collected as exploratory variables for safety analyses.
- Treatment information: Record the specific treatment program received by the subject, including the name of the drug, dosage, treatment cycle and so on.
- Symptoms and signs: Record any symptoms and signs that the subject experienced during the study period, including the severity and duration of the symptoms.
- Examination and test results: Record all relevant laboratory tests and imaging results.
- Health-related questionnaires: Record the results of health-related questionnaires completed by the subjects, including quality of life assessment, psychological status assessment, etc.

The CRF form was filled out offline, by trained researchers at each follow-up visit. To ensure the accuracy and completeness of the data, the researchers recorded the subjects’ information in detail during the follow-up visits and collected the CRF forms immediately after the follow-up visits. All CRF forms were double-checked within 24 hours of completion to ensure the accuracy and completeness of the data.

During the data entry phase, data in the CRF tables were imported into the study database by manual entry. During the entry process, each piece of data was entered by two independent data entry clerks and cross-validated to minimize entry errors. After data entry was completed, the data management team performed further quality control of the database, including data cleansing, logic checking, and consistency validation to ensure the accuracy and completeness of all data.

Multiple imputation using chained equations will be performed under the missing at random (MAR) assumption, with 20 imputed datasets created based on baseline characteristics and observed outcome data. Sensitivity analyses will be conducted to assess the robustness of results under different missing data assumptions, including best-case and worst-case scenarios. Complete case analysis will be presented as a secondary analysis.

For outliers, they are identified through visualization tools (e.g., box-and-line diagrams) and the values are removed or corrected as appropriate. In addition, data are regularly checked for completeness, consistency and scope to ensure data quality. All data processing was documented in detail so that other researchers could replicate the research process. Through these measures, this study ensured the high quality of the data and the reliability of the findings.

### Screening and baseline assessments

Each participant must undergo designated medical examinations and counseling within a two-week period before the trial commences.

- The baseline characteristics of the patients include gender, age, smoking status, and underlying diseases as well as the treatments, with a focus on respiratory conditions like chronic obstructive pulmonary disease (COPD) and interstitial lung diseases (ILDs).
- Lung cancer characteristics, including its anatomical site, dimensions, and TNM classification.
- Tumor molecular characteristics: Driver mutation status (EGFR, ALK, ROS1, KRAS, etc.) will be recorded if available from prior routine testing. This information will be used for exploratory analyses of potential associations between mutation status and RILI risk, as well as to document any prior or subsequent exposure to targeted therapies (TKIs) that may influence pneumonitis risk.
- Physical assessments include height, weight, vital signs, and respiratory status, with particular emphasis on auscultation for crackles.
- Blood tests, including complete blood count (CBC), serum biochemistry, coagulation function, C-reactive protein, and blood sedimentation.
- Chest CT and pulmonary function assessment.

### Investigational product

The investigatory medication, nintedanib, in capsules will be supplied by CSPC NBP Pharmaceutical Co., Ltd. Each capsule consists of 150 mg of the active ingredient. The prescribed dosage is 150 mg per administration, taken twice daily. The capsules should be ingested with food and water to minimize gastrointestinal side effects associated with nintedanib. It is important to swallow the whole capsule without chewing or crushing it due to the bitter taste of the product. Diarrhea is another prevalent adverse event that can be managed using traditional antidiarrheal drugs. If the adverse events persist or are intolerable, the dosage will be temporarily lowered to 100 mg per dose, twice daily until the participant can tolerate the symptoms. This approach aims to mitigate the occurrence of adverse events while ensuring the participant’s comfort and safety.

The initial administration will occur 1–2 weeks before CCRT to establish a consistent drug concentration in the bloodstream during treatment and maintain it for a duration of 12 weeks. The first dosing phase ends at week 12. From weeks 13–24, treatment will follow the principle of patient voluntarism, allowing participants to decide whether to continue or discontinue nintedanib based on their tolerance and preference. This voluntary period was designed to accommodate patient autonomy and reflect real-world clinical practice, given the lack of established evidence on the optimal duration of prophylactic nintedanib in this setting. Of note, the primary endpoint (incidence of grade ≥2 RILI) is assessed within 6 months after radiotherapy initiation, with the voluntary period commencing at week 12; therefore, the primary outcome is determined largely before the voluntary period begins.

The decision to discontinue drug treatment lies with the physician, who may do so based on the circumstances. Nintedanib therapy will be ceased permanently if severe adverse events occur, such as liver dysfunction, jaundice, severe hypersensitivity, and photosensitivity. It is important to be cautious when prescribing nintedanib to patients with mild hepatic impairment (Child-Pugh A) [23]. The recommended dosage for these individuals is 100 mg twice daily, with doses spaced approximately 12 hours apart, and should be taken with food.

### Anti-tumor therapy

Following the guideline recommendations, patients participating in this study will receive an anti-tumor treatment regimen that combines concurrent chemoradiotherapy with immunotherapy. Anti-tumor treatments are as follows.

### Radiotherapy

Definition related to tumor target area: outlining in CT simulation scan, outlining the target area and organs at risk. Gross tumor volume-primary tumor (GTVp) is the primary lung tumor after induction therapy. Gross Tumor Volume-Lymph Node (GTVnd) is the metastatic lymph node. The CTV includes the GTVp and the GTVnd externally placed 5 mm and includes the ipsilateral hilar as well as the mediastinal lymphatic drainage area that is involved before the induction therapy. On top of the above CTV, 5 mm of externalization was used to form the planning target volume (PTV). 5 mm of externalization of GTVp + GTVnd was used to form planning gross target volume (PGTV). The target area needed to be reviewed and modified for approval by at least one supervising physician and above senior physician. Tumor target area prescription dose: 95% PTV 60Gy/2Gy/30F. Critical organ limits: bilateral lung V20 < 28%, bilateral lung mean dose (MLD) <17 Gy; spinal cord PRV maximum dose (Dmax) <45 Gy; esophageal mean dose (Dmean) ≤34 Gy; cardiac V30 < 40%, V40 < 30.

### Chemotherapy and immunotherapy

Two cycles of chemotherapy administered every 3 weeks (Q3W) are required to include either cisplatin or carboplatin, along with one of the specified agents such as etoposide, vinorelbine, a taxane (docetaxel/paclitaxel), pemetrexed. The study proposes the use of immunoconsolidation therapy as a viable treatment option to improve the anti-cancer response in patients after completing chemoradiotherapy. This therapy will be administered within 90 days post-chemoradiotherapy to target the adapted population.

### Evaluation and Intervention in RP

#### Diagnosis of RP.

The diagnosis of RP relies on clinical suspicion and radiological findings after ruling out other lung pathologies, and will be assessed during follow-up by a multidisciplinary team. To ensure consistency and minimize bias, the primary endpoint (grade ≥2 RILI) will be determined by a blinded independent central review committee comprising two radiologists and one pulmonologist. Prior to study initiation, all reviewers will undergo centralized training on CTCAE v5.0 criteria using a standardized assessment manual. Each case will be independently reviewed by two committee members from different centers; disagreements will be adjudicated by a third senior expert to reach a consensus grade for the primary endpoint. Reviewers will have access to anonymized chest CT images and essential clinical information but will be blinded to treatment allocation and site-reported outcomes. For ambiguous cases, reviewers may request additional masked clinical information or consult the adjudication committee, and such cases will be flagged for sensitivity analyses.

The severity of RP will be scored according to the Common Terminology Criteria for Adverse Events version 5.0 (CTCAE V.5.0) [24], which categorizes symptoms and imaging results into five different grades. This standardized approach allows for consistent and objective assessment of RP severity in patients. The grade will be scored at the maximal level in cases that experience RP. The predominant symptoms in acute lung injury are dyspnoea and dry cough. Occasional fever typically presents as a mild symptom, while high fever may indicate the presence of co-infectious pneumonitis. Chronic radiation fibrosis (RF) is a slowly developing respiratory condition that may present as respiratory insufficiency. Physical symptoms can manifest as normal physical examination findings or as the presence of pleural rub, moist rales, and signs of consolidation.

Chest imaging, specifically baseline and follow-up lung CT scans, plays a crucial role in diagnosing and grading lung conditions. These imaging techniques are essential for monitoring changes in the lungs over time and assessing the severity of the disease. In the acute phase, typically occurring 4–8 weeks post-RT, CT scans can reveal exudative alterations characterized by multiple small patchy or flock-shaped ground glass density shadows within the irradiation field. These shadows have fuzzy edges and indistinct boundaries with the surrounding lung tissue. Such changes are indicative of RILI during the early phase following CRRT. The appearance of these exudative alterations on CT images reflects the inflammatory response in the irradiated lung tissue. In the consolidation phase following CRRT, CT images may reveal irregular high-density consolidations in the area that received radiation, not following the typical lung anatomy, along with signs of partial air bronchial. This phase typically happens 2–3 months after CRRT. In the fibrotic stage following CRRT, CT images may reveal density enhancement shadows in the irradiation field, characterized by clear boundaries and various shapes such as grid, cord, or patchy shapes. These shadows are accompanied by thickened pleura, reduction in lung volume and hilum, thinning of ipsilateral vascular texture, and a compensatory increase in contralateral lung volume. Overall, the fibrotic stage typically occurs around 6 months after CRRT and is characterized by specific changes in CT images, including density enhancement shadows with distinct shapes and boundaries, along with alterations in pleura thickness, lung volume, hilum size, vascular texture, and contralateral lung volume.

Radiation pneumonitis (RP) and immune checkpoint inhibitor-related pneumonitis (CIP) will be distinguished based on: (1) Temporal relationship: RP typically occurs 2–6 months post-radiotherapy; CIP can occur at any time during or after ICI treatment. (2) Radiologic features: RP presents with sharp borders confined to the radiation field; CIP is often bilateral, multifocal, with indistinct borders extending beyond the field. (3) Relationship to radiation field: RP changes are predominantly within the field; CIP changes frequently occur outside the field or are diffusely distributed. (4) Exclusion of other causes: Infection, tumor progression, and other drug-induced pneumonitis will be ruled out. All cases will be reviewed by a multidisciplinary team. If distinction is unclear, attribution will be recorded as “mixed/indeterminate” for sensitivity analyses.

#### Treatment of RP.

Glucocorticoid therapy involves the timely, appropriate, and personalized administration of hormones. This approach aims to optimize the therapeutic benefits while minimizing potential side effects [25]. Grade 1 RP typically does not necessitate intervention, with frequent observation being the primary approach. Oral prednisone at 0.5–1.0 mg/(kg/day) is recommended for patients with grade 2 RP with significant symptoms. Improvement and stabilization achieved post-treatment for 2–4 weeks warrants a gradual reduction in dosage by 5–10 mg/(kg/day) per week over a period of 4–12 weeks.

In patients with grade ≥3 RP, it is recommended to administer dexamethasone or methylprednisolone intravenously at an equivalent dose of 1–4 mg/(kg/day). The hormone dosage should be tapered gradually after improvement and stabilization of symptoms like cough and respiratory difficulty, typically within 1–2 weeks of treatment. It is important to carefully monitor patients receiving intravenous dexamethasone or methylprednisolone for grade ≥3 RP to ensure appropriate dosing and response. The dosage reduction program should be tailored to each individual based on the initial hormone dosage and disease conditions to gradually decrease the dosage. This individualized approach should continue until a lower dosage is reached. If the disease remains stable or improves to grade ≤2, transition to oral prednisone and taper the dosage gradually. If the disease is still in stage 3–4, it is recommended to adjust the hormone dosage accordingly. However, the effectiveness of a higher dosage is constrained. This implies that increasing the hormone dosage may not significantly improve treatment outcomes in advanced stages of the disease.

### Additional treatments

Additional interventions include the management of hormonal side effects, particularly in cases of high-dose hormone therapy. Additionally, proton pump inhibitors are utilized to safeguard the stomach lining while undergoing treatment. Supplementation with calcium and vitamin D is recommended for patients on extended glucocorticoid therapy to reduce the likelihood of developing osteoporosis. These treatments aim to address the potential adverse effects of hormonal therapy, minimize gastric damage, and reduce the risk of bone-related complications in patients undergoing glucocorticoid treatment.

Symptomatic patients with grade 2 and 3–4 radiation pneumonitis (RP) are at risk of developing pulmonary infections. In cases where infection is suspected, empirical anti-infective therapy is administered. Short-term administration of low-dose sulphonamide is recommended for preventing Pneumocystis carinii infections in high-risk populations, particularly in patients receiving high-dose hormones. These approaches aim to prevent and manage infections in vulnerable patients with RP. Symptomatic treatment involving antitussive and sputum medications is appropriate for patients with significant symptoms. Oxygen therapy is provided to patients experiencing hypoxia to increase the oxygen levels in the blood. Patients presenting with airway obstruction undergo nebulization to widen and moisten the airway. This treatment aims to improve airflow and alleviate symptoms associated with airway constriction.

### Post-treatment

The follow-up period will be half year. All participants will be followed up 1 month after radiotherapy and every 3 months thereafter, or as needed clinically. Routine follow-up will include the assessment of clinical symptoms and quality of life assessment, blood test, chest CT, pulmonary function evaluation. Also, the tumor’s disease status is assessed during follow-up visits, specifically at the end of radiotherapy, the thirth month, and sixth month. These time points are of particular interest for evaluation. The 1-year time point serves as an additional observation period for data collection. The data collected at the 1-year time point will be comparable to that collected at the 6th month time point. This additional time point allows for further insights to be gathered regarding the study outcomes.

### Assessments

George’s Questionnaire and the Health Status Questionnaire SF-36 were used to assess the health status of the subjects [26]. The symptoms and the impact of symptoms on daily life will be evaluated using the Patient’s Overall Impression of Disease Severity questionnaire (see as supl. materials) [27]. Imaging assessment of both radiation pneumonitis (RP) and the effectiveness of tumor therapy is crucial in this study.

Spirometry measurements will be performed according to ATS/ERS 2019 guidelines [28]. Force vital capacity (FVC) and carbon monoxide diffusion capacity (DLCO) will be assessed using standardized spirometry equipment at sites. FVC is deemed stable if the results fluctuate within ± 5% of the baseline level. An increase of more than 5% indicates improvement, while a decrease of more than 5% indicates deterioration. In the context of DLCO measurements, stability is defined as a variation of less than ± 10% from the baseline value. Improvement is noted when there is an increase of more than 10%, whereas deterioration is observed with a decrease of more than 10%.

Safety parameters mainly include the assessment of adverse events and safety laboratory parameters. The main focus of our study is on the gastrointestinal adverse reactions associated with nintedanib, such as diarrhea, nausea, vomiting, abdominal pain, decreased appetite, weight loss, and increased levels of liver enzymes. These reactions have been reported in published literature and are of particular interest in patients receiving nintedanib.

### Endpoints

The primary endpoint is the incidence of grade 2 or higher RP in the full analysis set and per-protocol set. Diagnosis and grading of RP will be confirmed after review by multidisciplinary senior physicians, including a radiation oncologist, a pulmonologist, and a radiologist. Medical history, physical parameters, chest CT scans, and previous radiotherapy will be evaluated at follow-up during study visits (Fig 1). RILI will be scored based on the CTCAE V.5.0 classifying symptoms and imaging findings will be used to classify into five grades (Table 2) [29] and the grade will be scored at the maximal level in cases who experience RILI. The case demonstrates that RILI will be managed according to the procedure described earlier.

**Table 2:** Incidence and grading criteria of radiation pneumonitis.

Grade	Clinical	Radiologic
1	Asymptomatic to minimally symptomatic	Ground glass opacities, less than 25% of lung involvement
2	Symptomatic requiring treatment, limitation of ADLs, but no oxygen requirement	Extensive ground glass opacities extending beyond therapy field with signs of no to minimal focal consolidation, involvement of 25–50% of lung
3	Symptoms with oxygen requirement	Clear evidence of focal consolidation with or without evidence of fibrosis, more than 50% involvement
4	Severe symptoms with persistent oxygen requirement or assisted ventilation	Dense consolidations, atelectasis, traction bronchiectasis with significant pulmonary volume loss
5	Death	

The progression from radiotherapy to the onset of RP will be recorded. The severity of RP and treatment outcomes will be assessed through symptom evaluation, imaging tests, and lung function measurements. A comparative analysis will be performed between the treatment group and the control group to assess the impact of investigational medicine on oncological treatment efficacy. Tolerability of treatments and occurrence of adverse drug reactions will also be compared between the two groups.

### Ethical approval

This study was approved by the Ethics Committee of the First Hospital of Zhangjiakou (No. 2024-IIT-007–03), Beijing Cancer Hospital (No. 2024YJZ166), Cancer Hospital Chinese Academy of Medical Sciences (No. 24/578–4858). Participants who meet the eligibility criteria have provided written informed consent and committed to actively take part in and complete the study, including all required follow-up procedures.

### Statistical analysis and determination of sample size

#### Methods of statistical analysis.

Statistical analyses will be conducted utilizing SPSS20.0. Measurement data will be presented as mean ± standard deviation (x ± s). The t test will be employed to compare means between two groups. The χ2 test will be utilized to compare rates between groups. The survival rate will be determined using the Kaplan-Meier method. Between-group comparisons will be performed using the log-rank test.

Serial pulmonary function tests (FVC%, FEV1%, DLCO%) will be analyzed using linear mixed-effects models with random intercepts and slopes to account for within-patient correlation over time. Time, treatment group, and their interaction will be included as fixed effects.

To address the potential confounding effects of treatment heterogeneity, the following statistical approaches will be implemented. First, the stratification factor (chemotherapy regimen) will be included as a covariate in the primary analysis model. Second, a multivariable logistic regression model will be performed to adjust for potential confounders, including chemotherapy regimen (categorized as etoposide-based, taxane-based, or pemetrexed-based), immunotherapy consolidation (yes/no), radiotherapy planning target volume (PTV, < 500 mL vs. ≥ 500 mL), and baseline pulmonary function. Third, prespecified subgroup analyses will be conducted to explore the treatment effect across different chemotherapy regimens and according to whether patients receive immunotherapy consolidation. These analyses will help assess the consistency of the treatment effect, although they will be exploratory in nature due to the limited sample size.

Exploratory analyses: In addition to the primary and secondary analyses, we will conduct exploratory analyses to examine potential associations between driver mutation status (EGFR, ALK, ROS1, KRAS, etc.) and the risk of RILI, as well as the impact of prior or subsequent TKI therapy on safety outcomes. These analyses are exploratory in nature and will be interpreted with caution given the limited sample size and the fact that mutation status is not a stratification factor. Results from these exploratory analyses will be used to generate hypotheses for future studies.

Given the exploratory nature of this study, secondary endpoints (OS, PFS, ORR, pulmonary function changes) will be analyzed without formal adjustment for multiplicity. All secondary analyses will be clearly labeled as exploratory, and p-values will be interpreted descriptively. Results will be presented with effect sizes and confidence intervals to aid interpretation. For survival outcomes, the confidence interval for the survival distribution will be computed using Greenwood’s formula, and between-group comparisons will be performed using the log-rank test. A significance level of p < 0.05 indicates statistical significance in the difference.

### Sample size calculation

Based on published literature, the incidence of grade ≥2 radiation pneumonitis in patients receiving concurrent chemoradiotherapy for locally advanced NSCLC is approximately 20% in the control group. We hypothesize that nintedanib prophylaxis will reduce this incidence to 5% in the treatment group. Using a two-sided χ² test with α = 0.05 and power = 80%, a sample size of 58 patients per group would be required. Accounting for a 10% dropout rate, we plan to enroll 66 patients in total (33 per group).

### Statistical analysis of the population

- Full Analysis Set (FAS): including all subjects who were randomised into the group. And those who have taken the drug at least once and have at least one follow-up record.
- Per-Protocol Set (PPS): all the cases that complied with the trial protocol, completed the visit plan, had good adherence, did not use prohibited drugs during the trial, completed the CRF and complied with the visit window, and the efficacy of the cases was statistically analysed.
- Safety Analysis Set (SS): All the cases that used the study drug at least once and have the safety evaluation data after the use of the drug constitute the safety dataset of this study. This data set was used to evaluate the safety of this study.

### Data collection and management

The research assistant will gather all clinical data and document it thoroughly in the predetermined electronic database. This process ensures accurate and comprehensive recording of clinical information for research purposes. The written informed consent forms will be securely stored in a designated location, with restricted access only granted to researchers handling the research data. This protocol ensures that confidentiality and data security are maintained throughout the research process.

To ensure participant retention and complete follow-up, we will implement multiple participant-centered support measures, including flexible visit scheduling, dedicated personnel for regular contact, and reminder systems. Even if participants discontinue the intervention, we will continue to collect key outcome data—such as primary efficacy endpoints, serious adverse events, survival status, and reasons for discontinuation—through alternative means such as telephone follow-up.

The final trial dataset will be accessible to the principal investigators, study statisticians, and relevant personnel directly involved in the analysis and reporting of this study. Additionally, access will be granted to regulatory authorities and the ethics committee for their respective oversight functions.

### Dissemination

The results will be shared at international academic conferences and published in peer-reviewed journals. Authorship will be granted to those who make substantial contributions to the conception/design, acquisition/analysis/interpretation of data, drafting or critical revision of the manuscript, and final approval of the version to be published.

### Patient and public involvement

Patients and the general public do not play a role in designing, executing, or measuring outcomes in the study.

## Discussion

The present study conducted a controlled trial to examine the effectiveness and safety of nintedanib in reducing RP in patients with unresectable stage II-III NSCLC. This study aims to investigate the preventive effects of medication on RP using an advanced medication approach. Positive outcomes could significantly benefit patient care and overall treatment success, especially for individuals at high risk during RP.

Nintedanib, a tyrosine kinase inhibitor, received approval in 2015 for treating IPF and specific ILDs. It is also approved in combination with docetaxel for treating adenocarcinoma non-small cell lung cancer (adeno-NSCLC) in some countries and regions. There have been attempts to investigate the potential use of nintedanib in preventing or treating RP due to the similarities in pathophysiology with pulmonary fibrosis. However, the limited published research findings have not yet yielded definitive conclusions. Nintedanib’s potential in preventing or treating RP was investigated in a mouse study, which showed positive results in reducing inflammation, alveolar debris, and resolving edema and vasculitis in thoracic irradiated animals. A recent clinical trial examined the efficacy of combining prednisone taper with nintedanib for treating RP, as compared to using prednisone taper alone [29]. The nintedanib group exhibited a lower incidence of pulmonary exacerbations of RP at one year compared to the control group, indicating the potential efficacy of nintedanib in managing RP [30]. Dy et al. conducted a study to assess the prophylactic impact of nintedanib on RP. The results indicated potential effectiveness, but the limited number of cases hindered definitive conclusions [31]. It is necessary to conduct studies with increased sample sizes to assess the efficacy of nintedanib in preventing and treating radiation pneumonitis.

The present research involves a six-month observation period, with additional data collection planned for the one-year duration from patients. The initial half-year is segmented into two 12-week phases. During the initial phase, participants in the experimental group receive nintedanib consistently. In the subsequent phase, individuals can choose to either continue or discontinue the drug for up to six months. By employing this design, researchers can evaluate the efficacy of nintedanib in preventing RP. Additionally, this approach enables an exploration of the course of medicine based on the participants’ natural inclinations. The study primarily investigates the incidence of grade 2 or higher RP and compares treatment outcomes of RP between patient groups. Additionally, the study also evaluates the effectiveness of nintedanib in treating lung cancer and assesses patient adherence and medication safety.

### Limitations

Our research has several limitations.

First, the unblinded trial design, while convenient, is susceptible to bias and subject expectancy effects, which may influence the reporting and interpretation of adverse events, as well as patient satisfaction and adherence. Patient-reported outcomes should be interpreted with particular caution given the open-label design.

Second, the sample size calculation assumed a 10% dropout rate. Should dropout exceed this, the study may be underpowered. Any positive findings will require confirmation in a larger, blinded, multicenter trial.

Third, the voluntary dosing period (weeks 13–24) introduces heterogeneity in drug exposure. However, the primary endpoint is assessed within six months of radiotherapy initiation, largely before this period begins. For secondary outcomes beyond week 12, sensitivity analyses stratified by actual treatment duration (≤12 weeks vs. > 12 weeks) will be conducted to account for exposure variability.

Fourth, the inclusion of multiple chemotherapy regimens and immunotherapy consolidation reflects real-world practice but may introduce confounding bias. We addressed this by stratifying randomization by chemotherapy regimen and adjusting for relevant covariates (immunotherapy consolidation, PTV, baseline pulmonary function) in the analyses. Nonetheless, residual confounding cannot be fully excluded, and subgroup analyses should be interpreted cautiously given the limited sample size.

Fifth, the absence of a centralized independent imaging core laboratory for RILI assessment is a limitation. To mitigate this, rigorous quality control measures were implemented as described in the Methods section. Future studies with larger sample sizes should incorporate centralized blinded review to enhance reliability.

Sixth, driver mutation status will be collected when available from routine testing, but this study is not powered to detect differences in RILI risk based on mutation status or prior TKI exposure. These data will be used for exploratory analyses and hypothesis generation only; definitive conclusions regarding interactions between mutation status, targeted therapy, and nintedanib’s preventive effect cannot be drawn. Future studies with larger samples and prospective molecular data collection are needed.

## Conclusions

In summary, the current study design aimed to assess the efficacy of nintedanib’s role on the prevention and treatment of RILI. These results have the potential to inform future interventions aimed at preventing RILI. Nevertheless, due to the constraints of the current study design, additional research is necessary to confirm the effectiveness of nintedanib in preventing RILI through a blinded study with a larger sample size.

## Supporting information

S1 File Study protocol. (PDF)

S2 File SPIRIT Checklist. (PDF)
