# Supplementary material for: Nintedanib in the prevention of radiation-induced lung injury in patients with unresectable stage II-III NSCLC: A protocol for a randomized, multicenter, control group study
Source: PLoS One. 2026 Sep 3;21(9):e0356205. doi: 10.1371/journal.pone.0356205 (PMC13541139; doi:10.1371/journal.pone.0356205)
Supplement: Supplementary file 1 [file pone.0356205.s001.pdf]

A prospective open-label clinical observation on the effectiveness and safety of Nintedanib in preventing radiation-induced lung injury after radiotherapy for NSCLC

Study protocol

Application organization:[redacted]

Principal Investigator:[redacted]

Documents Number:[redacted]

Version/Date:[redacted]

Abstract

**Study Name**	A prospective open-label clinical observation on the effectiveness and safety of Nintedanib in preventing radiation-induced lung injury after radiotherapy for NSCLC
**Inclusion criteria**	All of the following criteria must be met:<br>✓ Age between 18 and 80 years at time of screening<br>✓ Histologically/cytologically confirmed diagnosis of NSCLC<br>✓ TNM staging of stage II or III according to the International Association for the Study of Lung Cancerstaging manual (8th edition)<br>✓ Unsuitable for or unable to tolerate surgery<br>✓ WHO performance status of 0 or 1 at randomization<br>✓ No previous radiation therapy<br>✓ Be able to swallow gelatin Capsules<br>✓ Suitable for completing pulmonary function tests<br>✓ Volunteered to participate in the study and sign an informed consent form
**Exclusion criteria**	If any of the following criteria are met, they are excluded:<br>✓ Expected life expectancy of <6 months at randomization<br>✓ Severe cardiovascular, cerebrovascular, neurological diseases, infections, epilepsy, active peptic ulcer, psychological disorders, etc<br>✓ Abnormal liver function (transaminases elevated to 2.5 times or more the upper limit of normal)<br>✓ Abnormal renal function (creatinine clearance <30 ml/min) or requiring dialysis<br>✓ Recent anticoagulation therapy or combination of deep vein thrombosis pulmonary embolism, heart attack and cerebral infarction, etc<br>✓ Antifibrotic therapy within 1 month<br>✓ Allergy or intolerance to therapeutic drugs and ingredients<br>✓ Pregnant or breastfeeding women
**Removal criteria**	✓ Prolonged treatment shedding exceeding 48 hours or treatment discontinuation<br>✓ Researchers decided that the patient was not a suitable candidate for radiotherapy as originally planned

**Determination of fallout**	✓ Discontinuation of the clinical trial by a subject who, for whatever reason, is unwilling/unable to continue with the trial and requests withdrawal from the trial<br>✓ Although not explicitly withdrawing from the trial, subjects no longer receive medication and testing and are dropped from the trial<br>✓ Difficulty in continuing the medication due to the occurrence of other complications or worsening of comorbidities, as judged by the investigator<br>✓ According to the researcher's judgment, there is a drug adverse reaction event that requires the termination of the trial<br>✓ Other conditions occur that make it difficult to continue the medication, as judgedby the investigator
**sample size**	66
**Study medication**	Nintedanib capsules
**dosage regimen**	For patients who meet the inclusion criteria but do not meet the exclusion criteria, they will be randomly divided into an experimental group and a control group in a 1:1 ratio. The experimental group received conventional treatment combined with nintedanib, while the control group received only conventional treatment.<br>The specific plan is as follows:<br>**Experimental group:**Conventional clinical treatment combined with nintedanib, nintedanib is given for the first time 1-2 weeks before radiotherapy, with a dose of 150mg twice a day for 12 consecutive weeks; The first stage of medication is completed until 24 weeks after the start of radiotherapy. During this stage, the study follows the principle of voluntary medication, and patients who are willing to continue taking medication remain unchanged.<br>**Control group**: Adopting clinical conventional treatment plan.
**Concomitant medication**	1. Prohibit the use of drugs that may prevent or treat fibrosis, including amifostine and thalidomide;<br>2. Avoid the simultaneous use of drugs that increase adverse reactions to nintedanib, including ketoconazole or erythromycin;

	3. Avoid taking drugs that may affect the efficacy of Nintedanib at the same time, including azathioprine, cyclosporine, mycophenolate mofetil, tacrolimus, rituximab, cyclophosphamide, or oral glycosides
**Treatment and Visit**	V1（Screening period, 1-2 weeks before radiotherapy）、V2（week 1 after the start of radiotherapy）、V3（week 3 after the start of radiotherapy）、V4（week 5 after the start of radiotherapy）、V5（week 11 after the start of radiotherapy）、V6（week 24 after the start of radiotherapy）
**Endpoints**	**Primary endpoint:**<br>①The incidence of grade 2 or higher RP ;<br>**secondary endpoint:**<br>①Comparing time to radiological lung injury occurrence;<br>②Pulmonary function changes after radiotherapy;<br>③Treatment effects in patients with RILI;<br>④The efficacy outcomes (OS, PFS, and ORR) in patients with NSCLC;<br>⑤Assess changes in lung function (DLco%, FVC%, and FEV1%) from baseline at different time points;<br>⑥The occurrence and treatment efficacy of radiation pneumonitis (RP).<br>⑦ The results reported by patients include changes in symptoms such as shortness of breath, cough, and fatigue, as well as the impact on daily life and overall health assessment at different time points.
**Safety evaluation**	**Safety**: Incidence of the most common adverse reactions related to the investigational drug (gastrointestinal events such as diarrhea, abdominal pain, nausea/vomiting);<br>**Tolerance**:Proportion of subjects with dose adjustment, treatment interruption, and cessation of treatment;

Contents

Abstract 1
abbreviation 5
1 Introduction 6
2 Objective 7
3 Design 7
3.1 Overall experimental design 7
3.2 sample size 8
4 Patient selection 9
4.1 Inclusion criteria 9
4.2 Exclusion criteria 9
4.3 Removal criteria 9
4.4 dropout and management 10
5 Treatment plans and experimental drugs 10
5.1 Treatment plans 10
5.2 Investigational drug 15
5.3 Dose adjustment and delay 15
5.4 Drug interactions 16
5.5 contraindication 16
5.6 Termination criteria for treatment 17
6 Clinical trial process 17
6.1 Screening period 17
6.2 Visit period（V2～V5） 17
6.3 Visit period（V6） 18
7 Endpoints 19
7.1 effectiveness evaluation 19
8 adverse event 19
8.1 definition 19
8.2 Observation and manage of Adverse Events 19
8.3 Evaluation of the correlation between adverse events and research drugs 20
8.4 Report of adverse events 21
9 Medical Ethics 21
10 statistical analysis 22
10.1 Statistical analysis methods 22
10.2 Statistical analysis of population 22
11 Quality Control and Quality Assurance 22
11.1 quality control 22
11.2 quality assurance 24
12 Responsibilities assumed by all parties 24
13 Preservation and Summary of Experimental Data 24
14 Expected progress of the experiment 24
15 References 25
16 Appendix 26
Appendix 1. Classification of Common Adverse Events (CTCAE_5.0 Version) 26

abbreviation

ADR	Adverse drug reactions
AE	adverse event
ALT	Alanine aminotransferase
AST	aspartate aminotransferase
BID	Twice a day
CRF	Case Report Form
CTCAE	Evaluation criteria for common adverse event terminology
FVC	Forced lung capacity
GCP	Quality Management Standards for Clinical Trials of Drugs
HRCT	high resolution CT
RP	radiation pneumonitis
SAE	Serious adverse events
SGRQ	St George's respiratory questionnaire
DLco%	Carbon monoxide dispersion capacity
FVC	Forced lung capacity
FEV1	First second forced expiratory volume
RIP	radiation pneumonitis
RIPF	Radiation-induced pulmonary fibrosis
NSCLC	Non small cell lung cancer

1 Introduction

The incidence rate and mortality of lung cancer rank first in all kinds of malignant tumors, of which surgical treatment is the first choice, while radiotherapy is considered to be an important means not suitable for surgery and further treatment after surgical treatment[1]。As the dose-response relationship of radiation therapy changes, the toxic side effects on normal tissues increase with the increase of radiation dose[2]， Among them, radiation-induced lung injury (radiation pneumonitis and radiation pulmonary fibrosis) is the most common and has the greatest impact on patients[3]。

Radiation pneumonitis is an inflammatory reaction caused by the damage of normal lung tissue in the radiation field after radiotherapy of lung cancer, esophageal cancer, breast cancer, malignant lymphoma, mediastinal tumor and other chest tumors. The incidence of this disease is 5% to 50%, the incidence of severe pneumonia is 10% to 20%, and the mortality rate is as high as 50%[4]。Radiation pneumonitis has the highest incidence in radiotherapy for lung cancer. Radiation induced lung injury mainly includes two manifestations: Radiation induced Pneumonitis（RIP) and Radiation induced Pulmonary Fibrosis（RIPF)[5-6]。RIP mainly occurs 2-6 months after radiotherapy, with significant changes in clinical manifestations. Mild cases may be asymptomatic, often accompanied by coughing, fever, and difficulty breathing. Severe cases can be life-threatening. The pathological manifestation is exudative inflammatory response in the acute phase.In terms of imaging, the early stage is mainly characterized by exudation, while the middle stage is mainly characterized by abnormalities, manifested as patchy shadows, bronchial signs, cord shadows, lung consolidation shadows, or honeycomb like changes limited to the irradiation area. A small number of patients may have changes outside the radiation area in addition to changes within the irradiation area[7-10]。RIRF develops relatively slowly and usually occurs six months to several years after radiotherapy. Clinical manifestations include varying degrees of respiratory distress. The pathological manifestation is fibrotic lesions. On imaging, the main changes are interstitial changes and fibrous hyperplasia, manifested as reduced lung volume, increased fibrous cord shadows, thickened interlobular septa, ipsilateral pleural thickening, and bronchial, hilar, and mediastinal traction displacement. Factors related to radiation pneumonitis include radiation dose, irradiated lung volume, baseline lung function, and concurrent use of chemotherapy or immunotherapy drugs.In addition to direct physical damage from radiation, its main mechanism is closely related to acute immune responses mediated by inflammatory mediators, involving a series of cytokines, inflammatory mediators, and the migration and repair of various cells.

At present, there is no particularly effective treatment for radiation-induced lung injury, which is mainly supported by high-dose corticosteroids, combined with oxygen therapy, tracheal dilation and other drugs for symptomatic treatment[11]， And do a good job in infection control and prevention, even so, the clinical treatment effect of radiation pneumonia is limited, so the most important means for radiation pneumonia at present is still early prevention. Previous studies have confirmed[12-15]， Radiation induced lung injury and idiopathic pulmonary fibrosis have similar pathological processes, and nintedanib can effectively treat idiopathic pulmonary fibrosis. Nintedanib is a small molecule tyrosine kinase inhibitor with anti fibrotic and anti-inflammatory activities. Nintedanib can inhibit various receptor tyrosine kinases (RTKs): platelet-derived growth factor receptors alpha and beta (PDGFR alpha, PDGFR beta), fibroblast growth factor receptors 1-3 (FGFR1-3), vascular endothelial growth factor 1-3 (VEGFR1-3), and Fms like tyrosine kinase-3 (FLT-3). Among them, FGFR, PDFGR, and VEGFR are related to the pathogenesis of idiopathic pulmonary fibrosis. Nintedanib can competitively bind to the adenosine phosphate (ATP) binding sites on these intracellular receptor kinase domains, tissue intracellular signaling, and inhibit fibroblast proliferation, migration, and transformation[16]。In animal models of fibrosis, Nintedanib exhibits anti-inflammatory, anti fibrotic, and vascular remodeling improving effects[17]， In summary, Nintedanib may play a role in preventing radiation pneumonitis during radiotherapy.

2 Objective

Evaluate the efficacy and safety of nintedanib in preventing local recurrence (RILI) in patients with unresectable stage II-III non-small cell lung cancer (NSCLC) undergoing concurrent chemoradiotherapy (CCRT).

3 Design

3.1 Overall experimental design

This study is a randomized, controlled, multicenter clinical trial evaluating the clinical efficacy, safety, and tolerability of Nintedanib in patients with lung cancer who have developed lung injury after radiation therapy. Eligible subjects will be randomly assigned to the experimental group and the control group in a 1:1 ratio.

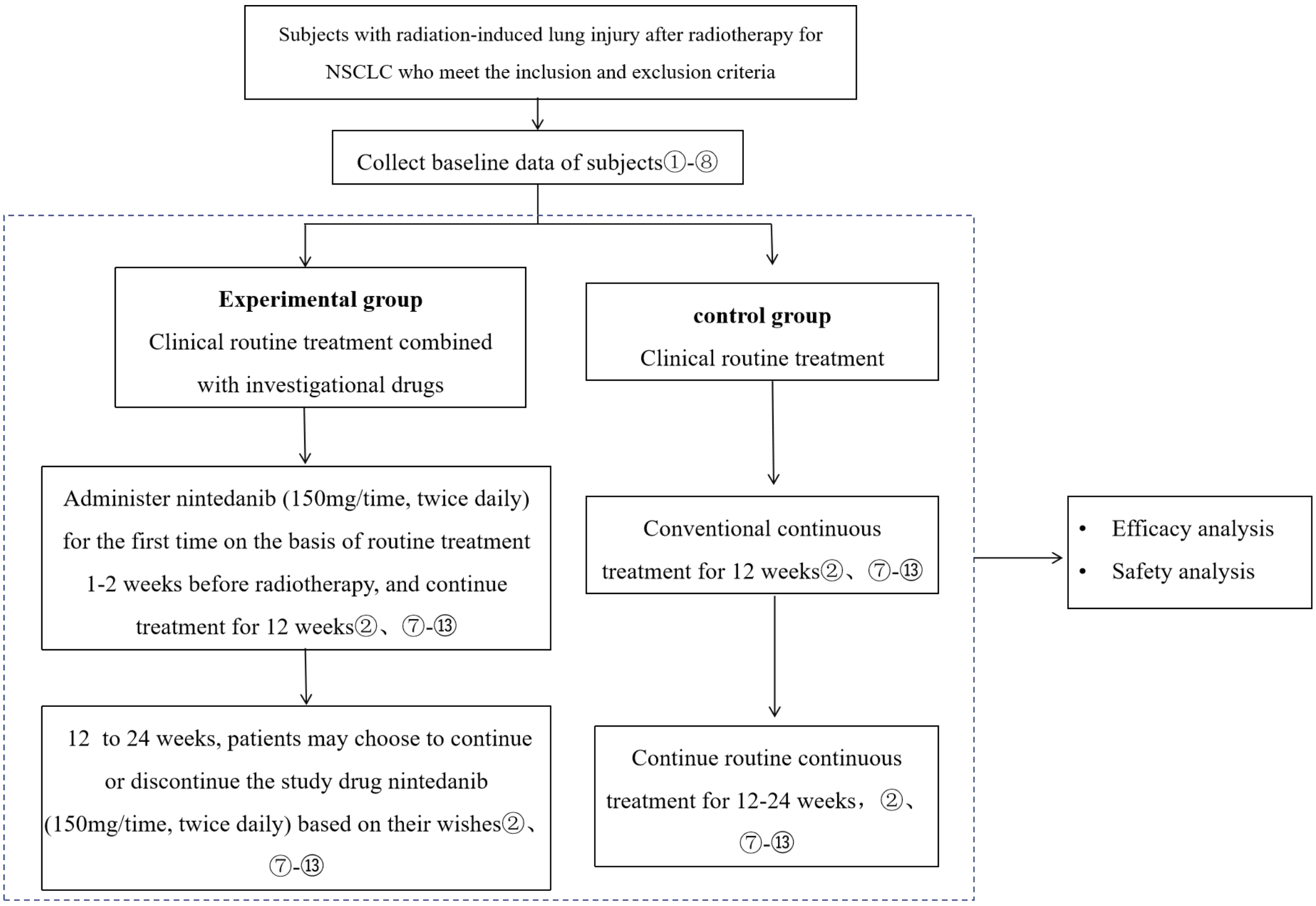

Annotation:

The numbers represent the data collection projects, which are:

① Basic data;

② Patient complaints (cough, difficulty breathing, etc.), symptoms (severity, duration), and signs;

③ Vital signs (body temperature, heart rate, blood pressure) and physical examination;

④ Medical history and surgical history;

⑤ Clinical diagnosis, clinical symptoms;

⑥ Blood routine, blood biochemistry;

⑦ Imaging examination results;

⑧ FEV_1, FVC (forced vital capacity);

⑨ The occurrence of grade 2 and above radiation pneumonitis;

⑩ Inquire and record adverse events and serious adverse events;

⑪ Treatment information: Inquire and record whether there is a dose adjustment, treatment interruption, or cessation of treatment;

⑫ Inquire and record accompanying treatments/medications;

⑬ Health-related questionnaire;

3.2 Sample size

Sample size was determined using the Non-Inferiority Tests for One Proportion method of

PASS15.0 software. The cut-off value (δ) was set at 34%, α at 0.025 (unilateral), and the degree of certainty (1-β) at 80%. This resulted in a sample size of 60 cases. The calculation was based on the specified parameters to ensure adequate statistical power. The software was utilized to determine the appropriate sample sizefor the study. The study enrolled 66 cases with a assumed subject shedding rate of 10%.

4 Patient selection

4.1 Inclusion criteria

All of the following criteria must be met:

- ✓ Age between 18 and 80 years at time of screening
- ✓ Histologically/cytologically confirmed diagnosis of NSCLC
- ✓ TNM staging of stage II or III according to the International Association for the Study of Lung Cancerstaging manual (8th edition)
- ✓ Unsuitable for or unable to tolerate surgery
- ✓ WHO performance status of 0 or 1 at randomization
- ✓ No previous radiation therapy
- ✓ Be able to swallow gelatin capsule
- ✓ Suitable for completing pulmonary function tests
- ✓ Volunteered to participate in the study and sign an informed consent form

4.2 Exclusion criteria

If any of the following criteria are met, they are excluded:

- ✓ Expected life expectancy of <6 months at randomization
- ✓ Severe cardiovascular, cerebrovascular, neurological diseases, infections, epilepsy, active peptic ulcer, psychological disorders, etc.
- ✓ Abnormal liver function (transaminases elevated to 2.5 times or more the upper limit of normal)
- ✓ Abnormal renal function (creatinine clearance <30 ml/min) or requiring dialysis
- ✓ Recent anticoagulation therapy or combination of deep vein thrombosis, Pulmonary embolism, heart attack and cerebral infarction, etc.
- ✓ Antifibrotic therapy within 1 month
- ✓ Allergy or intolerance to therapeutic drugs and ingredients
- ✓ Pregnant or breastfeeding women

4.3 Removal criteria

- ✓ Prolonged treatment shedding exceeding 48 hours or treatment discontinuation

- ✓ Researchers decided that the patient was not a suitable candidate for radiotherapy as originally planned

4.4 dropout and management

Determination of dropout: Regardless of when or why the patient withdraws, as long as the clinical trial observation has not been completed, it is considered a dropout case.

Those who meet one of the following criteria will be considered as dropouts:

- ✓ Discontinuation of the clinical trial by a subject who, for whatever reason, is Unwilling/unable to continue with the trial and requests withdrawal from the trial
- ✓ Although not explicitly withdrawing from the trial, subjects no longer receive Medication and testing and are dropped from the trial
- ✓ Difficulty in continuing the medication due to the occurrence of other Complications or worsening of comorbidities, as judged by the investigator
- ✓ Experience an adverse drug reaction event that warrants discontinuation of the trial, as judged by the investigator
- ✓ Other conditions occur that make it difficult to continue the medication, as judged by the investigator

Handling of dropout cases: When a subject falls out, the researcher must fill in the reason for dropout in the CRF and try to contact the subject as much as possible to complete the items that can be evaluated, record the last medication time for analysis of its efficacy and safety, and keep the CRF for future reference.

Note: If a patient terminates the chemotherapy regimen and study drug treatment prescribed in this study prematurely due to serious adverse events, revocation of informed consent, disease complications or comorbidities, disease progression, or other circumstances, completing two cycles of study drug treatment is also considered a completed case. Do not handle cases of detachment. If the researchers believe that the patient needs to change the initial chemotherapy regimen, the patient will no longer be followed up.

5 Treatment plans and experimental drugs

5.1 Treatment plans

For patients who meet the inclusion criteria but do not meet the exclusion criteria, they will be randomly divided into an experimental group and a control group in a 1:1 ratio.

The specific plan is as follows:

Experimental group: The initial administration will occur 1-2 week before RT to establish a consistent drug concentration in the bloodstream during treatment and maintain it for a duration of 12 weeks. The first dosing phase ends at week 12, and from weeks 12 to 24,

treatment will follow the principle of patient voluntarism. During the voluntary period, patients will have the autonomy to decide on their continued participation in the treatment regimen。

Control group: Adopt clinical routine treatment.

The decision to discontinue drug treatment lies with the physician, who may do so based on the circumstances. Nintedanib therapy will be ceased permanently if severe adverse events occur, such as liver dysfunction, jaundice, severe hypersensitivity, and photosensitivity. It is important to be cautious when prescribing nintedanib to patients with mild hepatic impairment (Child-Pugh A) . The recommended dosage for these individuals is 100 mg twice daily, with doses spaced approximately 12 hours apart, and should be taken with food.

Anti-tumor therapy

Following the guideline recommendations, patients participating in this study will receive an anti-tumor treatment regimen that combines concurrent chemoradiotherapy with immunotherapy. Anti-tumor treatments are as follows

Radiotherapy

Definition related to tumor target area: outlining in CT simulation scan, outlining the target area and organs at risk. Gross tumor volume-primary tumor (GTVp) is the primary lung tumor after induction therapy. Gross Tumor Volume-Lymph Node (GTVnd) is the metastatic lymph node. The CTV includes the GTVp and the GTVnd externally placed 5mm and includes the ipsilateral hilar as well as the mediastinal lymphatic drainage area that is involved before the induction therapy. On top of the above CTV, 5mm of externalization was used to form the planning target volume (PTV). 5mm of externalization of GTVp+GTVnd was used to form planning gross target volume (PGTV). The target area needed to be reviewed and modified for approval by at least one supervising physician and above senior physician. Tumor target area prescription dose: 95% PTV 60Gy/2Gy/30F. Critical organ limits: bilateral lung V20 <28%, bilateral lung mean dose (MLD) <17 Gy; spinal cord PRV maximum dose (Dmax) <45 Gy; esophageal mean dose (Dmean) ≤34 Gy; cardiac V30 <40%, V40 <30.

Chemotherapy and immunotherapy

Two cycles of chemotherapy administered every 3 weeks (Q3W) are required to include either cisplatin or carboplatin, along with one of the specified agents such as etoposide, vinorelbine, a taxane (docetaxel/paclitaxel), pemetrexed. The study proposes the use of immunoconsolidation therapy as a viable treatment option to improve the anti-cancer response in patients after completing chemoradiotherapy. This therapy will be administered within 90 days post-chemoradiotherapy to target the adapted population

Evaluation and Intervention in RP

Diagnosis of RP

The diagnosis of RP relies on clinical suspicion or radiological findings after ruling out other lung pathologies like pre-existing conditions and pulmonary infection.

During follow-up visits, medical history, physical parameters, chest CT scans, and past radiotherapy will be assessed by multidisciplinary senior physicians, including a pulmonologist, a radiation oncologist, and a radiologist. This will be done at personal visits when they can be scheduled.

The severity of RP will be scored according to the Common Terminology Criteria for Adverse Events version 5.0 (CTCAE V.5.0) , which categorizes symptoms and imaging results into five different grades. This standardized approach allows for consistent and objective assessment of RP severity in patients. The grade will be scored at the maximal level in cases that experience RP. The predominant symptoms in acute lung injury are dyspnoea and dry cough. Occasional fever typically presents as a mild symptom, while high fever may indicate the presence of co-infectious pneumonitis. Chronic radiation fibrosis (RF) is a slowly developing respiratory condition that may present as respiratory insufficiency. Physical symptoms can manifest as normal physical examination findings or as the presence of pleural rub, moist rales, and signs of consolidation.

Chest imaging, specifically baseline and follow-up lung CT scans, plays a crucial role in diagnosing and grading lung conditions. These imaging techniques are essential for monitoring changes in the lungs over time and assessing the severity of the disease. In the acute phase, typically occurring 4–8 weeks post-RT, CT scans can reveal exudative alterations characterized by multiple small patchy or flock-shaped ground glass density shadows within the irradiation field. These shadows have fuzzy edges and indistinct boundaries with the surrounding lung tissue. Such changes are indicative of RILI during the early phase following RT. The appearance of these exudative alterations on CT images reflects the inflammatory response in the irradiated lung tissue. In the consolidation phase following RT, CT images may reveal irregular high-density consolidations in the area that received radiation, not following the typical lung anatomy, along with signs of partial air bronchial. This phase typically happens 2-3 months after RT. In the fibrotic stage following RT, CT images may reveal density enhancement shadows in the irradiation field, characterized by clear boundaries and various shapes such as grid, cord, or patchy shapes. These shadows are accompanied by thickened pleura, reduction in lung volume and hilum, thinning of ipsilateral vascular texture, and a compensatory increase in contralateral lung volume. Overall, the fibrotic stage typically occurs around 6 months after RT and is characterized by specific changes in CT images, including density enhancement shadows with

distinct shapes and boundaries, along with alterations in pleura thickness, lung volume, hilum size, vascular texture, and contralateral lung volume.

Treatment of RP

Glucocorticoid therapy involves the timely, appropriate, and personalized administration of hormones. This approach aims to optimize the therapeutic benefits while minimizing potential side effects. Grade 1 RP typically does not necessitate intervention, with frequent observation being the primary approach. Oral prednisone at 0.5-1.0 mg/(kg-d) is recommended for patients with grade 2 RP with significant symptoms. Improvement and stabilization achieved post-treatment for 2-4 weeks warrants a gradual reduction in dosage by 5-10 mg/(kg-d) per week over a period of 4-12 weeks.

In patients with grade ≥3 RP, it is recommended to administer dexamethasone or methylprednisolone intravenously at an equivalent dose of 1-4 mg/(kg-d). The hormone dosage should be tapered gradually after improvement and stabilization of symptoms like cough and respiratory difficulty, typically within 1-2 weeks of treatment. It is important to carefully monitor patients receiving intravenous dexamethasone or methylprednisolone for grade ≥3 RP to ensure appropriate dosing and response. The dosage reduction program should be tailored to each individual based on the initial hormone dosage and disease conditions to gradually decrease the

dosage. This individualized approach should continue until a lower dosage is reached.

If the disease remains stable or improves to grade ≤2, transition to oral prednisone and taper the dosage gradually. If the disease is still in stage 3-4, it is recommended to adjust the hormone dosage accordingly. However, the effectiveness of a higher dosage is constrained. This implies that increasing the hormone dosage may not significantly improve treatment outcomes in advanced stages of the disease.

Additional treatments

Additional interventions include the management of hormonal side effects, particularly in cases of high-dose hormone therapy. Additionally, proton pump inhibitors are utilized to safeguard the stomach lining while undergoing treatment. Supplementation with calcium and vitamin D is recommended for patients on extended glucocorticoid therapy to reduce the likelihood of developing osteoporosis. These treatments aim to address the potential adverse effects of hormonal therapy, minimize gastric damage, and reduce the risk of bone-related complications in patients undergoing glucocorticoid treatment.

Symptomatic patients with grade 2 and 3-4 radiation pneumonitis (RP) are at risk of developing pulmonary infections. In cases where infection is suspected, empirical anti-infective therapy is administered. Short-term administration of low-dose sulphonamide is recommended

for preventing Pneumocystis carinii infections in high-risk populations, particularly in patients receiving high-dose hormones. These approaches aim to prevent and manage infections in vulnerable patients with RP. Symptomatic treatment involving antitussive and sputum medications is appropriate for patients with significant symptoms. Oxygen therapy is provided to patients experiencing hypoxia to increase the oxygen levels in the blood. Patients presenting with airway obstruction undergo nebulization to widen and moisten the airway. This treatment aims to improve airflow and alleviate symptoms associated with airway constriction.

Post-treatment

The follow-up period will be half year. All participants will be followed up 1 month after radiotherapy and every 3 months thereafter, or as needed clinically. Routine follow-up will include the assessment of clinical symptoms and quality of life assessment, blood test, chest CT, pulmonary function evaluation. Also, the tumor's disease status is assessed during follow-up visits, specifically at the end of radiotherapy, the thirth month, and sixth month. These time points are of particular interest for evaluation. The 1-year time point serves as an additional observation period for data collection. The data collected at the 1-year time point will be comparable to that collected at the 6th month time point. This additional time point allows for further insights to be gathered regarding the study outcomes.

Assessments

George's Questionnaire and the Health Status Questionnaire SF-36 were used to assess the health status of the subjects. The symptoms and the impact of symptoms on daily life will be evaluated using the Patient's Overall Impression of Disease Severity questionnaire (see as supl. materials) . Imaging assessment of both radiation pneumonitis (RP) and the effectiveness of tumor therapy is crucial in this study.

Spirometry measurements will be performed according to ATS/ERS 2019 guidelines [26]. Force vital capacity (FVC) and carbon monoxide diffusion capacity (DLCO) will be assessed using standardized spirometry equipment at sites. FVC is deemed stable if the results fluctuate within ± 5% of the baseline level. An increase of more than 5% indicates improvement, while a decrease of more than 5% indicates deterioration. In the context of DLCO measurements, stability is defined as a variation of less than ± 10% from the baseline value. Improvement is noted when there is an increase of more than 10%, whereas deterioration is observed with a decrease of more than 10%.

Safety parameters mainly include the assessment of adverse events and safety laboratory parameters. The main focus of our study is on the gastrointestinal adverse reactions associated with nintedanib, such as diarrhea, nausea, vomiting, abdominal pain, decreased appetite, weight

loss, and increased levels of liver enzymes. These reactions have been reported in published literature and are of particular interest in patients receiving nintedanib.

5.2 Investigational drug

Investigational drug:YILUODA®（Nintedanib Ethyl Sulfonate Soft Capsules）

Specifications: Calculated based on C31H33N5O4（1）150mg（2）100 mg;

Approval number:150mg:National Medical Products Administration Approval Letter H20213164;100mg:National Medical Products Administration Approval Letter H20213163;

Storage conditions: Please store below 25 ℃. Please store in a safe location that children cannot reach or see!

Packaging: Solid medicinal high-density polyethylene bottle; 30 pills/bottle.

Manufacturer: Enbipu Pharmaceutical Co., Ltd. of CSPC

Validity period: 36 months

Usage and dosage: The recommended dosage of this product is 150 mg each time, twice a day, with an interval of approximately 12 hours between administration.

This product should be taken with food and whole capsules should be taken with water. This product has a bitter taste and should not be chewed or crushed for consumption. The effect of chewing or crushing capsules on the pharmacokinetics of Nintedanib is not yet clear.

If a dose of medication is missed, the recommended dose should be continued at the next scheduled medication time point and the missed dose should not be taken again. It should not exceed the recommended maximum daily dose of 300 mg.

Patients with mild liver injury (Child Pugh A) should use with caution. The recommended dose of Nintedanib is 100mg twice daily, approximately 12 hours apart, and taken with food.

5.3 Dose adjustment and delay

If applicable, in addition to symptomatic treatment, the treatment of adverse reactions of this product (see instructions [Precautions], [Adverse Reactions]) may include reducing the dose and temporarily interrupting administration until specific adverse reactions are relieved to a level that allows for continued treatment. The standard recommended dose (150 mg twice daily) or a reduced dose (100 mg twice daily) can be used to restart treatment with this product. If the patient cannot tolerate 100 mg twice a day, the treatment with this product should be stopped.

Elevated liver enzymes may require adjustment of dosage or interruption of treatment. When the liver enzymes return to baseline values, the dose of this product can be reduced again (100mg twice daily) and then increased to the full dose (150mg twice daily).

Use with caution in patients with mild liver injury (Child Pugh Class A). When managing adverse reactions, consideration should be given to interrupting or stopping treatment.

5.4 Drug interactions

（1）**P-glycoprotein（P-gp）**

Nintedanib is a substrate of P-gp. If administered in combination with this product, P-gp potent inhibitors (such as ketoconazole or erythromycin) can increase exposure to nintedanib. In these cases, the patient's tolerance to Nintedanib should be closely monitored. Dealing with adverse reactions may require interrupting, reducing the dosage, or stopping the treatment with this product (see [Dosage and Administration]).

Strong inducers of P-gp, such as rifampicin, carbamazepine, phenytoin, and St. John's wort, can reduce exposure to nintedanib. Consideration should be given to selecting alternative combination drugs that have no P-gp induction effect or minimal induction effect.

（2）Food

Suggest taking this product together with food (see pharmacokinetics)。

（3）Cytochrome (CYP) enzyme

The biotransformation of Nintedanib relies only minimally on the CYP pathway. In preclinical studies, nintedanib and its metabolites (free acid portion BIBF 1202 and its glucuronide compound BIBF 1202 glucuronide) did not inhibit or induce CYP enzymes. Therefore, it is believed that the possibility of drug interaction with Nintedanib based on CYP metabolism is low.（4）Combined administration with other drugs

The combination administration of Nintedanib and Bosentan did not alter the pharmacokinetics of Nintedanib.

The combined administration of Nintedanib and oral hormonal contraceptives did not have a significant correlation with the pharmacokinetics of oral hormonal contraceptives. nintedanib has pH dependent solubility characteristics, and its solubility increases in acidic environments with $pH<3$. However, in clinical trials, combined administration with proton pump inhibitors or histamine H2 antagonists had no effect on the exposure level (trough concentration) of nintedanib.

（4）Anticoagulant

Nintedanib is a VEGFR inhibitor that may increase the risk of bleeding. Patients receiving full dose anticoagulant therapy should be closely monitored to prevent bleeding, and anticoagulant therapy should be adjusted if necessary.

5.5 Contraindication

This product is contraindicated for patients who are known to be allergic to Nintedanib,

peanuts, soybeans, or any excipients of this product. This product is contraindicated during pregnancy (detailed information can be found in the instructions for "Medication for Pregnant and lactating Women" and "Pharmacology and Toxicology").

5.6 Termination criteria for treatment

In this study, when Nintedanib (YILUODA®) is terminated, it is called "termination of treatment research", and the specific situation is as follows:

①The patient requests to withdraw themselves or their legal representative requests to withdraw;

②Adverse events related or unrelated to treatment during treatment force treatment termination;

③Patient death;

④Violating the protocol regulations and affecting the evaluation of research results;

⑤Researchers believe that patients should discontinue treatment studies.

If the termination of the treatment study is caused by adverse reactions, it is necessary to track the adverse reactions until they are properly resolved and the condition stabilizes.

6 Clinical trial process

This experiment includes:V1（Screening period, 1-2 weeks before radiotherapy）、V2（week 1 after the start of radiotherapy）、V3（week 3 after the start of radiotherapy）、V4（week 5 after the start of radiotherapy）、V5（week 11 after the start of radiotherapy）、V6（week 24 after the start of radiotherapy）

6.1 Screening period

Qualified enrolled patients are required to undergo the following examinations during the screening period/baseline:

(1) Basic patient information (age, gender, date of birth, ethnicity, height, weight, history of drug allergies, family history);

(2) Patient complaints (cough, difficulty breathing, etc.);

(3) Vital signs (body temperature, heart rate, blood pressure) and physical examination;

(4) Medical history and surgical history;

(5) Clinical diagnosis, clinical symptoms;

(6) Blood routine, blood biochemistry;

(7) FEV_1、FVC;

6.2 Visit period（V2～V5）

Timely collect, record, and report information and data of subjects, while retrieving and counting trial research supplies and new trial documents and materials generated from clinical trials, to ensure the timeliness, consistency, completeness, reliability, and accuracy of trial

research data and information. Complete the following evaluation and record it in CRF.

(1) Patient's chief complaint (cough, difficulty breathing, etc;

(2) Symptoms and signs: Record any symptoms and signs that the subjects experience during the study period, including the severity and duration of the symptoms;

(3) Treatment information: Record the specific treatment plan received by the subject, including drug name, dosage, treatment period, etc.

(4) The incidence of grade 2 and above radiation pneumonitis;

(5) Inquire and record adverse events and serious adverse events;

(6) Inquire and record whether there are dose adjustments, treatment interruptions, and cessation of treatment;

(7) Inquire and record accompanying treatments/medications;

(8) FEV1、FVC;

(9) Blood routine, blood biochemistry, and imaging examination results;

(10) Health related questionnaire: Record the results of the health-related questionnaire filled out by the subjects, including quality of life assessment, psychological state assessment, etc

6.3 Visit period（V6）

Timely collect, record, and report information and data of subjects, while retrieving and counting trial research supplies and new trial documents and materials generated from clinical trials, to ensure the timeliness, consistency, completeness, reliability, and accuracy of trial research data and information. Complete the following evaluation and record it in CRF.

(1) Patient's chief complaint (cough, difficulty breathing, etc);

(2) Symptoms and signs: Record any symptoms and signs that the subjects experience during the study period, including the severity and duration of the symptoms;

(3) Treatment information: Record the specific treatment plan received by the subject, including drug name, dosage, treatment period, etc.

(4) The incidence of grade 2 and above radiation pneumonitis;

(5) Inquire and record adverse events and serious adverse events;

(6) Inquire and record whether there are dose adjustments, treatment interruptions, and cessation of treatment;

(7) Inquire and record accompanying treatments/medications;

(8) FEV1、FVC;

(9) Blood routine, blood biochemistry, and imaging examination results;

(10) Health related questionnaire: Record the results of the health-related questionnaire filled out by the subjects, including quality of life assessment, psychological state assessment, etc

7 Endpoints

7.1 effectiveness evaluation

(1)The primary endpoint is the incidence of grade 2 or higher RP;

(2)The secondary endpoint:

①Comparing time to radiological lung injury occurrence;

②Pulmonary function changes after radiotherapy;

③Treatment effects in patients with RILI;

④The efficacy outcomes (OS, PFS, and ORR) in patients with NSCLC;

⑤Assess changes in lung function (DLco%, FVC%, and FEV1%) from baseline at different time points;

⑥The occurrence and treatment efficacy of radiation pneumonitis (RP).

8 Adverse event

8.1 definition

Adverse event: refers to an adverse medical event that occurs after a patient or clinical trial subject receives a drug, but it does not necessarily have a causal relationship with the treatment.

Serious Adverse Event: A reaction caused by the use of a drug during a clinical trial that results in one of the following adverse conditions: (1) death; (2) Endangering life; (3) Carcinogenesis, teratogenicity, and birth defects; (4) Causing significant or permanent human disability or damage to organ function; (5) Causing hospitalization or prolonged hospitalization time; (6) Causing other important medical events that, if left untreated, may lead to the above-mentioned situations.

Adverse drug reactions: Refers to harmful rather than expected reactions that occur during the normal use of drugs at prescribed doses, but are causally related to drug use. In clinical trials of a new drug or drug for a new use, all harmful reactions that are not expected and have a causal relationship with the use of the drug should be considered as adverse drug reactions when the therapeutic dose has not been determined.

8.2 Observation and manage of Adverse Events

Observation and recording: Researchers should carefully observe any adverse events that occur in subjects during the clinical study period, require subjects to truthfully report changes in their condition after medication, and avoid suggestive questioning. Pay attention to adverse reactions or unexpected toxic side effects (including symptoms, signs, and laboratory tests) while observing the therapeutic effect. Regardless of whether the adverse event is related to the investigational drug or not, it should be recorded in detail in the CRF form, including the time of

occurrence, symptoms, signs, degree, duration, laboratory examination indicators, treatment methods, process, results, follow-up time, etc. The situation of concomitant drug use should also be recorded in detail to analyze the correlation between the adverse event and the investigational drug. The record should be signed and dated.

Medical treatment of subjects: When adverse reactions are detected, researchers can take necessary measures based on the condition, such as adjusting dosage, temporarily interrupting medication, and deciding whether to terminate the trial. Take necessary measures in case of serious adverse events to protect the safety of the subjects.

Follow up of serious adverse events: If a serious adverse event occurs that is unrelated to the investigational drug, does not cause serious harm to the patient, and has no significant impact on the main efficacy indicators, the patient can still be followed up on schedule and continue the trial; All other situations should be exited.

All adverse events should be tracked and observed to normal or almost normal to ensure the safety of the subjects. Follow up methods can be selected based on the severity of adverse reactions, including hospitalization, outpatient visits, home visits, phone calls, communication, and other forms.

8.3 Evaluation of the correlation between adverse events and research drugs

Researchers should conduct a comprehensive analysis based on the specific circumstances of the adverse events that occurred in the subjects, as well as their medical history, comorbidities, and medication history, to determine the relationship between the adverse events and the medication. Researchers should conduct causal analysis on the symptoms experienced by subjects during medication in order to evaluate the possible association between adverse events and the investigational drug. The factors considered in causal analysis include the following five aspects:

(1) Is there a reasonable chronological relationship between the start of medication and the occurrence of suspected adverse drug reactions (ADRs);

(2) Whether the suspected ADR conforms to the known ADR type of the drug (in accordance with literature);

(3) Can the suspected ADR be explained by concomitant medication, previous medication, the patient's clinical condition, or the effects of other therapies (other explanations);

(4) Whether the suspected ADR disappears or alleviates after discontinuation or reduction of medication (disappearance after discontinuation);

(5) After being exposed to the same medication again, does the suspected ADR reappear (and then reproduce).

The determination of causal relationship between adverse events and drugs: Based on the above five indicators, the causal relationship is analyzed into five levels: definitely related, possibly related, possibly unrelated, unrelated, and unable to determine.

- ✓ Unable to determine: Adverse events and investigational drugs cannot be determined;
- ✓ Unrelated: Adverse events are not related to the investigational drug;
- ✓ Possible unrelated: Adverse events are more likely to be explained by other factors;
- ✓ Possible correlation: Adverse events may be related to the investigational drug, and other explanations are not convincing;
- ✓ Definitely related: The adverse event has been listed as a possible adverse reaction and there is no reason to use other explanations.

8.4 Report of adverse events

In the process of clinical research, regardless of whether the adverse events are related to the investigational drug, researchers should truthfully and detailedly fill out the adverse event record form, recording the clinical manifestations, occurrence time, severity, duration, measures taken, and outcomes of the adverse events. And record in detail the situation of combined medication, in order to analyze the correlation between adverse events and the investigational drug.

Any serious adverse events that occur during the trial, regardless of whether they are related to the investigational drug, should be promptly treated by the researcher. The ethics committee of the clinical trial responsible unit and the sponsor should be notified by phone/fax within 24 hours. The sponsor should promptly investigate the serious adverse events that occur with the researcher, take necessary measures to ensure the safety of the subjects, and notify other researchers involved in clinical trials involving the same drug.

Researchers must fill out the 'Serious Adverse Event Report Form', which should record in the original data when, how, and to whom serious adverse events were reported.

Reporting channels for serious adverse events:

organization	Contact	telephone	fax

9 Medical Ethics

Clinical trials must adhere to the ethical, moral, and scientific principles outlined in the Helsinki Declaration (2000 edition) and the Chinese Good Clinical Practice (GCP). Before the clinical trial begins, the trial protocol must be reviewed and approved by the ethics committee of

the leading unit, and the approval opinion must be signed before implementation. During the trial, any modifications to the trial protocol must be approved by the ethics committee before implementation. Any serious adverse events that occur during the trial must be reported to the ethics committee.

10 statistical analysis

10.1 Statistical analysis methods

Statistical analyses will be conducted utilizing SPSS20.0. Measurement data will be presented as mean ± standard deviation (x ± s). The t test will be employed to compare means between two groups. The χ2 test will be utilized to compare rates between groups. The survival rate will be determined using the Kaplan-Meier method.

Between-group comparisons will be performed using the log-rank test. The confidence interval (CI) for the survival distribution will be computed using Greenwood's formula. Greenwood's formula will be used to calculate the CI of the survival distribution. A significance level of $p < 0.05$ indicates statistical significance in the difference

10.2 Statistical analysis of population

Full Analysis Set，FAS:Including all randomized participants. And those who have taken medication at least once and have at least one follow-up record.

Per-Protocol Set，PPS:Statistical analysis will be conducted on the efficacy of all cases that comply with the trial protocol, complete the visit plan, have good compliance, did not use prohibited drugs during the trial period, completed the CRF requirements, and met the visit window requirements.

Safety Analysis Set，SS:All cases that have used the investigational drug at least once and have safety evaluation data after medication constitute the safety dataset of this study. This dataset is used to evaluate the safety of this study.

11 Quality Control and Quality Assurance

11.1 Quality control

(1) Quality control measures in the laboratory

Establish unified experimental testing indicators, standard operating procedures, and quality control procedures in each participating clinical trial hospital laboratory.

(2)Requirements for participants

Corresponding professional expertise, qualifications, and research abilities.

(3)Training for participants

Through pre clinical trial training, participants will have a full understanding and recognition of the clinical trial protocol and its specific indicators (such as research objectives,

inclusion and exclusion criteria, prohibited drugs, etc.).

The objective indicators specified should be checked according to the time, place, and method specified in the plan.

Pay attention to observing adverse events or unexpected toxic side effects, and track and observe them. The description of subjective symptoms should be objective and should not induce or prompt.

The case report form should be filled out in a timely, truthful, and accurate manner to ensure that all conclusions in clinical trials are derived from raw data.

(4)Measures to ensure compliance of subjects

The participating doctors should patiently explain to the subjects, so that they fully understand and cooperate with the experiment.

(5)other

Inform the subjects of possible adverse reactions to the investigational drug, and immediately notify the doctor or hospital by phone if any adverse reactions occur.

11.2 Quality assurance

To ensure the quality of the trial, the researcher shall develop a clinical research plan before the formal trial begins. Each research center must manage research drugs in accordance with standard operating procedures, including acceptance, storage, distribution, and retrieval. It is not allowed to use research drugs with non participants of this clinical trial, and it is necessary to ensure that the drugs used are only for the subjects of this clinical trial.

The main researcher is responsible for the implementation of the entire experiment and researching solutions to issues related to the experiment.

The sponsor shall appoint a monitor for this trial to ensure that the data recorded and reported in the clinical trial is accurate, true, complete, and error free, and to ensure that the trial follows the approved protocol, the Good Clinical Practice for Drugs, and relevant regulations.

12 Responsibilities assumed by all parties

After the clinical trial unit completes the trial summary, researchers can publish papers and participate in domestic and international academic conferences; When publishing a paper, it should be clearly stated in the appropriate section of the paper: the research and development unit and production unit of the experimental drug.

The project funding unit is responsible for monitoring and inspecting the clinical trial, and providing the researchers with trial fees and qualified test drugs.

13 Preservation and Summary of Experimental Data

After the completion of the experiment, all case report forms shall be filled out and reviewed by the supervisor and the research leaders of each unit in accordance with the requirements of data management in this protocol.

The data management and statistical analysis unit is responsible for establishing a clinical data database and conducting unified data management; Statistical analysts compile all data for statistical analysis and write a statistical analysis report; The responsible unit for the trial has completed the Clinical Trial Summary.

14 Expected progress of the experiment

2024.02-03， Complete the design and finalization of the clinical trial protocol and its accompanying documents.

2024.03-04，The plan shall be submitted to the ethics committee for approval, and relevant content shall be modified according to the opinions of the ethics committee.

2023.04-05， Obtain approval from the ethics committee and complete preclinical preparations.

2024.05-12，Complete the enrollment and follow-up of experimental cases.

2025.01-03，Data processing and data summarization mainly involve inputting, verifying, analyzing, and summarizing the data obtained from clinical trials.

15 References

1. Marks LB,Bentzen SM,Deasy JO,et al.Radiation dose-volume effects in the lung［J].Int JR adiat Oncol Biol Phys,2010,76(3) :70-76.

2. Jenkins P, Welsh A. Computed tomography appearance of early radiation injury to the lung: correlation with clinical and dosimetric factors[J]. Int J Radiat Oncol Biol Phys,2011;81(1):97-103.

3. Jenkins P, Watts J. An improved model for predicting radiation pneumonitis incorporating clinical and dosimetric variables [J]. Int J Radiat Oncol Biol Phys, 2011; 80(4):1023

4.Lvhua Wang,et al. Chinese Journal of Radiation Oncology . 2015;24(1):4-9

5. Shannon VR, et al. Support Care Cancer. 2020 Dec;28(12):6145-6157.

6. Skeoch S, et al. J Clin Med. 2018 Oct 15;7(10):356.

7 Su Q, et al. Front Immunol. 2019 Feb 4;10:108.

8.DEＲEＲ A,FＲEY B, FIETKAU Ｒ,et al． Immune-modulating properties of ionizing radiation: Rationale for the treatment of cancer by combination radiotherapy and immune checkpoint inhibitors［J］. Cancer Immunology Immunotherapy,2016,65 (7) : 779-786．

9.HODGE JW， AＲDIANI A， FAＲSACI B，et al． The tipping point for combination therapy: cancer vaccines with radiation， chemotherapy， or targeted small molecule inhibitors［J］.Seminars in Oncology,2012,39(3) : 323-339．

10.FＲEY B,ＲUBNEＲ Y,KULZEＲ L,et al． Antitumor immune responses induced by ionizing irradiation and further immune stimulation［J］. Cancer Immunology Immunotherapy，2014，63(1) : 29-36.

11. GＲAY JE,VILLEGAS A,DANIEL D,et al.Three-year overall survival with durvalumab after chemoradiotherapy in stage III NSCLC-update from PACIFIC［J］.Journal of Thoracic Oncology,2020,15(2) : 288-293.

12.Mazzei ME et al. Ther Adv Respir Dis. 2015 Jun;9(3):121-9.

13. Ackermann M et al. Angiogenesis 2017;20:359–72.2. Huang J, et al. Ann Rheum Dis 2017;76:1941–8.3. Redente EF et al. Am J Physiol Lung Cell Mol Physiol 2018;314:L998–1009. 4. Lee HY, et al. Exp Lung Res .2017;43(4-5):187-196.

14. Flaherty KR, et al. N Engl J Med. 2019;381(18):1718-1727.

15. Wells AU, et al. Lancet Respir Med. 2020 May;8(5):453-460.

16. Fang W, Huang Y, Gan J, et al. Nintedanib Effect in Osimertinib-Induced Interstitial Pneumonia[J]. Journal of Thoracic Oncology, 2020, 15(3): e34-e35.

17. Yamakawa H, Oba T, Ohta H, et al. Nintedanib allows retreatment with atezolizumab of combined

non-small cell lung cancer/idiopathic pulmonary fibrosis after atezolizumab-induced pneumonitis: a case report[J]. BMC Pulmonary Medicine, 2019, 19(1): 1-5.

16 Appendix

Appendix 1. Classification of Common Adverse Events (CTCAE_5.0 Version)

gastrointestinal diseases Grade					
**adverse event**	**1**	**2**	**3**	**4**	**5**
diarrhea	Compared to baseline, the frequency of bowel movements increased by less than 4 times per day; Mild increase in discharge from the fistula opening	Compared to baseline, the frequency of bowel movements increased by 4-6 times per day; Moderate increase in discharge from the stoma; Daily life activities limited by tools	Compared to baseline, the frequency of bowel movements increased by ≥ 7 times per day; Need hospitalization treatment; Compared to baseline, there was a severe increase in the discharge from the stoma; Limited self-care daily living activities	Endangering life; Urgent treatment is required	death
nausea	Decreased appetite without accompanying changes in eating habits	Reduced oral intake without significant weight loss, dehydration, or malnutrition	Insufficient oral intake of energy and water; Need nasal feeding, total parenteral nutrition, or hospitalization	-	-
vomit	No intervention is needed	Outpatient intravenous fluid replacement; Medical intervention is required	Need nasal feeding, total parenteral nutrition or hospitalization treatment	Life-threatening situation	death
Elevated blood bilirubin	>1.5 times the upper limit of normal value (baseline value is normal); Greater than 1-1.5 times the baseline value	Greater than 1.5~3.0 times the upper limit of normal values (baseline values are normal); Greater than 1.5~3.0 times the baseline value	Greater than 3.0 to 10 times the upper limit of normal values (baseline values are normal); Greater than 3.0~10 times the baseline value	Greater than 10 times the upper limit of normal values (baseline values are normal); More than 10 times the	-

	(abnormal baseline value)	(abnormal baseline value)	(abnormal baseline value)	baseline value (abnormal baseline value)	
Elevated alanine aminotransferase	>Three times the upper limit of the normal value (baseline value is normal); 1.5 to 3.0 times the baseline value (if the baseline value is abnormal)	3-5 times the upper limit of the normal value (normal baseline value), greater than 3.0-5.0 times the baseline (if the baseline value is abnormal)	5-20 times (if the baseline value is normal); 5-20 times greater than the baseline value (if the baseline value is abnormal)	More than 20 times the upper limit of the normal value (if the baseline value is normal); Greater than 20 times the baseline value (if the baseline value is abnormal)	-
Elevated aspartate aminotransferase	More than 3 times the upper limit of the normal value (baseline value is normal); Greater than 1.5 to 3.0 times the baseline value (abnormal baseline value)	3-5 times greater than the upper limit of normal values (baseline values are normal); Exceeding 3.0 to 5.0 times the baseline value (abnormal baseline value)	5.0-20.0 times greater than the upper limit of normal values (baseline values are normal); Greater than 5.0 to 20.0 times the baseline value (if the baseline value is abnormal)	20.0 times greater than the upper limit of normal values (baseline values are normal); Greater than 20.0 times the baseline value (if the baseline value is abnormal)	-
Elevated alkaline phosphatase	More than 2.5 times the upper limit of the normal value (baseline value is normal); 2-2.5 times the baseline value (abnormal baseline value)	2.5 to 5.0 times greater than the upper limit of normal values (baseline values are normal); Greater than baseline 2.5 to 5.0 times the value (if the baseline value is abnormal)	5.0-20.0 times greater than the upper limit of normal values (baseline values are normal); Greater than 5.0 to 20.0 times the baseline value (if the baseline value is abnormal)	20.0 times greater than the upper limit of normal values (baseline values are normal); Greater than 20.0 times the baseline value (if the baseline value is abnormal)	-
